# Computerized vs. Paper-Pencil Assessment of Cognitive Change following Acute Ischemic Stroke

**DOI:** 10.4172/2329-6895.1000317

**Published:** 2016-12-01

**Authors:** Maude-Marie Gagnon, Robert Laforce

**Affiliations:** 1 Département des Sciences Neurologiques, CHU de Québec-Université Laval, Québec, Canada; 2 Clinique Interdisciplinaire de Mémoire, Département des Sciences Neurologiques, CHU de Québec-Université Laval, Québec, Canada; 3 Faculty of Medicine, Laval University, Québec, Canada

**Keywords:** Computerized testing, Paper-pencil testing, Cognition, Stroke

## Abstract

**Importance:**

Cognitive impairment is common among patients with stroke and early recognition can optimize patient care.

**Objective:**

To determine the validity of computerized cognitive testing in an adult population with acute ischemic stroke.

**Design:**

Validation study comparing computerized vs paper-pencil assessments at two time points three months apart in a stroke unit.

**Main outcome:**

Correlation analyses between computerized (using CogState Brief Battery) and paper-pencil testing (using the Montreal Cognitive Assessment) both at study entry and follow-up visits.

**Results:**

We found moderate to strong significant correlations between the two instruments at study entry and follow-up sessions. Executive dysfunctions were the main cognitive changes. Test-retest correlations were strong.

**Conclusion and Relevance:**

The CogState Brief Battery is a valid alternative for clinicians who wish to measure cognitive skills following acute ischemic stroke. Limitations of computerized testing are discussed.

## Background

Cognitive impairment is common among patients with stroke, particularly in elderly population with comorbid conditions [1]. Indeed, two thirds of stroke survivors experience cognitive changes and many develop dementia [2]. These changes have a significant impact on long-term outcome and appear to be associated with a weaker rehabilitation potential as well as increased risk of death, disability [3], depression and a lower quality of life [4,5]. Early recognition and monitoring of cognitive changes in stroke is necessary to optimize patient care and this can guide therapy and rehabilitation strategies [6,7].

To this date, evidence shows that cognitive assessment using the Montreal Cognitive Assessment (MoCA) in cerebrovascular disease is valid, covering a wide range of functions including executive functions and memory [8–13] with the exception of processing speed. Education level has been consistently reported to impact the total score on MoCA [14]. The feasibility of the MoCA in ischemic stroke is significantly altered by aphasia. As such, computerized testing may provide an alternative to these limitations. For example, computerized testing allows measurement of attention, processing speed, working memory and visual learning. Furthermore, it offers test-retest sessions without practice effect [15–19].

Among the computerized assessment devices available, research has shown that the CogState Brief Battery is valid in different clinical populations including dementia [20, 21] and concussion [22,23]. It has also been used for the assessment of attention in acute stroke and correlated with neuropsychological measures at 3 months [24].

To our knowledge, no study has yet compared the MoCA and the CogState in the acute phase and at 3-months post-stroke. Therefore, the goal of this research was to compare computerized vs paper-pencil cognitive assessment methods in patients with confirmed ischemic stroke both within the acute and later stages of an ischemic stroke. We hypothesized that computerized assessment results would be moderately correlated with the total score on MoCA in patients with ischemic stroke both at study entry and at 3-months follow-up.

## Methods

This study was approved by the Ethics Review Board of the local research center (Centre Hospitalier Universitaire de Québec). All participants provided written informed consent. The authors were responsible for the study design and conduct.

### Participants

Participants were men and women aged over 18 years old presenting with an acute ischemic stroke, confirmed by brain magnetic resonance imaging (MRI). Exclusion criteria were requirement of an interpreter, severe deficits in vision, hearing, language or motor impairment of the dominant arm preventing completion of the cognitive assessment, past history of dementia or severe intellectual disability, a diagnosis of acute delirium, an acute medical condition (such as myocardial infarction or sepsis), a living address off town and contra-indications to brain MRI (e. g. pacemaker).

### Design

**Figure 1:**
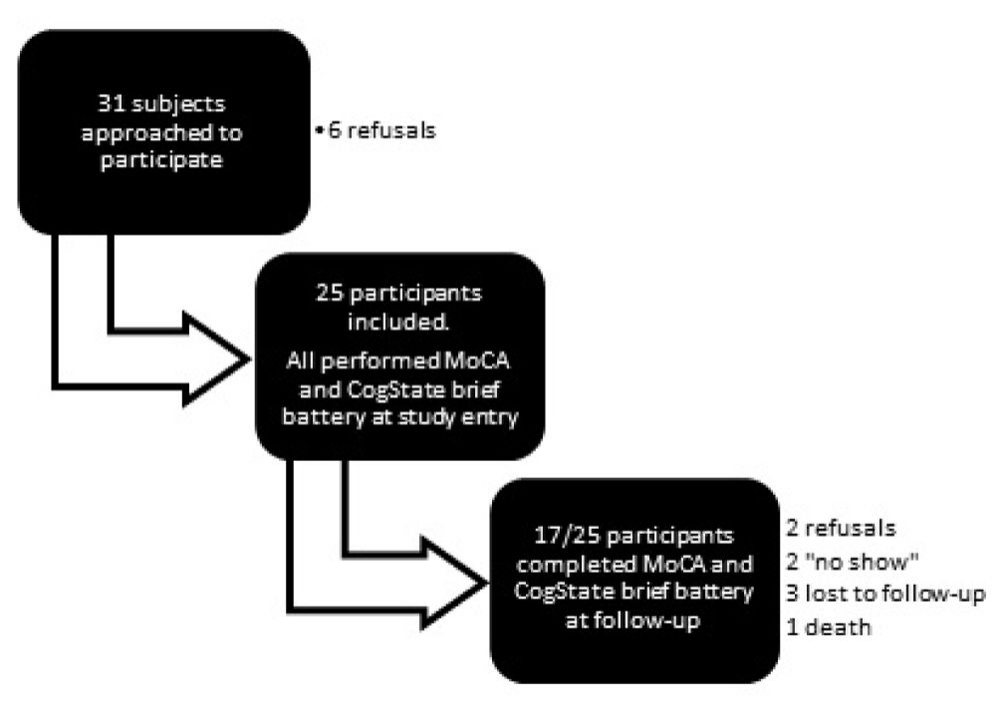
Study flowchart.

We conducted this validation study between July 2014 and October 2015 in the acute stroke unit of our tertiary neurological care center. The trial consisted of 3 phases: screening and recruitment, a first testing session within 2 weeks of the stroke and a follow-up testing session approximately 3 months post-stroke (Figure 1).

### Screening and Recruitment

Medical files and brain MRI were screened for each participant (by one of the author). Demographic data (gender, age), history of disease, medication use, clinical presentation of stroke including language, vision, motor, sensory and cerebellar impairments as well as signs and symptoms suggestive of delirium (such as disorientation, psychomotor agitation or hallucinations) were collected (Table 1). Localisation and lateralisation of stroke as well as presence of at least one lobar cerebral microbleed (CMB) [25] and leukoaraiosis [26,27] were gathered (Table 2). All brain MRIs were reviewed (by one of the author) to confirm eligibility and to grade leukoaraiosis using Fazekas’s scale. Susceptibility weighted imaging (SWI) sequences were also reviewed to validate presence and localisation of CMBs.

### Cognitive Measures

Computerized testing was performed using the CogState Brief Battery (or Cognigram). For each task, instructions were provided on the computer screen, followed by a playing card presented face down in the center of the screen. After a short interval, the card turned face up and the participants were required to respond (with “yes” or “no”) based on questions that varied for each task. It included 4 different tasks. The “Detection” task was a reaction time test of psychomotor function that required the participant to respond as quickly as possible when the central card was turned face up. The “Identification” task measured visual attention and required the participant to respond differently if the face up card was red or black. The “One card learning” task was a visual recognition learning task that assessed visual recognition memory and attention and required the participant to determine whether the face up card had appeared in the current task previously. The last task was the “One back” which assessed working memory and attention and required participants to determine whether the face up card was the same as the preceding card [16]. Performance measures were recorded for each trial on each task with speed in milliseconds and accuracy in percentage of correct and incorrect answers. Results were divided in 2 sections, each scored out of 200 points: “Attention and psychomotor function” which included the “Detection” and “Identification” tasks, and “Learning and working memory function” which included the “One card learning” and “One back” tasks. A score under 81 was considered abnormal, as standardized by CogState.

Paper-pencil test consisted of the MoCA, a well-known written screening measure which includes various items (i.e. orientation, visuospatial ability, executive functions, language, memory, attention and abstraction) scored out of 30 [28]. A score under 26 on the MoCA is usually considered abnormal.

Administration of the MoCA and CogState Brief Battery was held in a quiet environment using standardized instructions. Participants were contacted by phone for scheduling of follow-up visit.

### Statistical Analysis

Pearson’s correlation coefficient tests were conducted both at initial and follow-up testing sessions using data from the MoCA total score (out of 30) and the CogState Brief Battery test scores (global score and sub-scores).

## Results

Twenty-five patients were tested at study entry and 17 patients at 3 months follow-up (Figure 1). Mean age was 67 years old (SD=11.54; range 44–87). Hypertension, dyslipidemia and diabetes were the main comorbidities. The mean level of education was 12 years (SD=2.96; range 7 to 20). A majority of patients presented significant neurological deficits (17/25, 68%) at study entry (motor or sensory impairments involving the dominant hand, persistent mild expressive aphasia in 6 patients, right quadranopsia or hemianopsia in 3 patients or incoordination (Table 1). Strokes more frequently involved the middle cerebral artery on the left side on brain MRI, leuroaraiosis was frequently described (20/23, 87%) whereas CMBs were rare (4/23, 17%). Cortical and subcortical atrophy was present in 15 patients (Table 2).

The mean MoCA score at study entry was 23/30 (SD=4.68; range 12 to 30), and the mean scores for CogState Brief Battery were 91/200 for both parts (attention and psychomotor function: SD=15.42, range 51 to 115; learning and working memory: SD=13.9, range 61 to 114). At follow-up, the mean MoCA score was 25/30 (SD=4.00; range 13 to 30), and the mean scores for CogState Brief Battery were 96 (SD=13.35; range 51 to 115) for “Attention and psychomotor function” and 97 (SD=7.60; range 61 to 114) for “Learning and working memory”.

Correlational analyses between performances on the MoCA and CogState Brief Battery are shown in Table 3. Pearson correlation coefficients show moderate to strong correlations, with a significant moderate correlation between the global scores. Of note, correlation strength was similar after adjusting for age, gender and education level. Finally, there was a strong test-retest correlation (0.76, p=0.0004) for both computerized and paper-pencil measures.

## Discussion

In this study, we aim to compare computerized vs paper-pencil cognitive assessment methods in patients with confirmed ischemic stroke, both within the acute and later stages of the process. We found a significant moderate correlation between the global scores on MoCA and CogState.

This was not influenced by age, gender or education level. Finally, both paper-pencil and computerized assessment techniques were associated with strong test-retest correlations.

At first glance, a computerized test which offers test-retest capabilities with no practice effect and is more feasible than many other cognitive assessments appeared interesting. However, we noted that some patients tended to lose interest in executing the task throughout testing likely as a result of repetitive patterns.

Other participants appeared anxious because of the computer-based format. Some patients clearly stated that the test was boring. On the other hand, the youngest participants appeared to appreciate the playful aspect of the computerized test, noticeably the playing cards and sound effects. In this population of patients with stroke, the same limitations in test execution were present for MoCA and CogState Brief Battery in terms of motor, sensory, visual and coordination deficits.

This study is flawed by several limitations. First, our gold standard remained a brief cognitive screening tool and ideally performances should have been compared to comprehensive neuropsychological tests. Another important limitation is the sample size, which is obviously very small. Moreover, there were many drop-outs, representing 32% of the total sample. Potential explanations include the study design, anxiety related to testing and lack of interest given the absence of a disease-modifying intervention. This is important to consider since it may reflect the difficulties faced by clinicians in performing cognitive assessments in this population. Our sample was composed of mildly disabled stroke patients and thus our findings lack generalisability. We excluded patients with severe acute stroke due to the fact that they could not complete the tasks. We found the MoCA to be equally flawed in patients with global aphasia, dominant hand paralysis or fluctuating levels of consciousness. The impact of language impairments was subjectively less pronounced with CogState but not totally absent. Participant indeed received written and oral instructions for every task. Administration of the computerized assessment was as time consuming as the MoCA and sometimes even longer. Finally, the CogState Brief Battery tended to categorize patients with abnormal MoCA as normal.

## Conclusion

Altogether, we found moderate to strong significant correlations between computerized (CogState Brief Battery) and paper-pencil (MoCA) testing both at study entry and follow-up visits. Executive dysfunctions were the main cognitive changes. Test-retest correlations were strong. Computerized testing is a valid alternative for clinicians who wish to measure cognitive skills following acute ischemic stroke.

## Figures and Tables

**Table 1:** Participants’ characteristics (n=25).

Age (years): Mean (S.D), range	67 (11.54), 44–87
Gender (% male)	17 (68%)
Level of education (years): Mean (S.D), range	12 (2.96), 7–20
**Medical history (%)**	
Hypertension	13 (52%)
Dyslipidemia	12 (48%)
Diabetes	8 (32%)
Previous stroke	4 (16%)
Epilepsy	2 (8%)
Coronary heart disease	5 (20%)
Atrial fibrillation/flutter	1 (4%)
Depression	1 (4%)
**Medication (%)**	
Active use of psychotropic medications	5 (20%): Zopiclone(2), temazepam(1), trazodone (1), quietiapine, mirtazapine and lorazepam(1)
**Neurological sequelae (%)**	
Visual impairment	3 (12%)
Motor impairment (dominant hand)	4 (16%)
Sensitive impairment (dominant hand)	3 (12%)
Language impairment	8 (32%)
Coordination impairment	3 (12%)
No patent neurological deficit	8 (32%)

**Table 2:** Brain imaging characteristics.

Stroke localization (%)	Column1
Anterior cerebral artery	1 (4%)
Middle cerebral artery	15 (60%)
Posterior cerebral artery	1 (4%)
≥ one large vessel	3 (12%)
Infratentorial	3 (12%)
Thalamic lacunar infarct	2 (8%)
**Stroke lateralization (%)**	
Left	13 (52%)
Right	9 (36%)
Bilateral	3 (12%)
Leukoaraiosis (%)*	
Absence	3 (13%)
Fazekas I	16 (70%)
Fazekas II	1 (4%)
Fazekas III	3 (13%)
Cerebral microbleeds (%)*	
Absence	19 (83%)
Lobar	2 (9%)
Alobar	2 (9%)
**Others (%)**	
Presence of atrophy	15/23 (65%)
Previous infarcts	9/23 (39%)
Lacunar infarcts	6/25 (24%)

* All brain MRI included T2/FLAIR as well as SWI sequences except for 2 patients who had only a DWI sequence available. Therefore, the * indicates when the sample size was n=23.

**Abbreviations:** DWI: Diffusion-Weighted Imaging; MRI: Magnetic Resonance Imaging; FLAIR: Fluid-Attenuated Inversion Recovery; SWI: Susceptibility-Weighted Imaging.

**Table 3:** Correlation analyses between MoCA and CogState brief battery scores (Pearson’s coefficients).

	At study entry (n=25)	P value	At follow-up (n=17)	P value
Global scores	0.40505	0.0446*	0.59966	0.0109**
Sub-scores(attention and psychomotor functions)	0.44078	0.0274*	0.42404	0.0898
Sub-scores(learning and working memory)	0.29795	0.148	0.65124	0.0046***

* p<0.05;

** p<0.01;

*** p<0.005
